# Fatty Acid Synthase as a Factor Required for Exercise-Induced Cognitive Enhancement and Dentate Gyrus Cellular Proliferation

**DOI:** 10.1371/journal.pone.0077845

**Published:** 2013-11-05

**Authors:** Nataliya E. Chorna, Iván J. Santos-Soto, Nestor M. Carballeira, Joan L. Morales, Janneliz de la Nuez, Alma Cátala-Valentin, Anatoliy P. Chornyy, Adrinel Vázquez-Montes, Sandra Peña De Ortiz

**Affiliations:** 1 Department of Biology, Metabolomics Research Center, University of Puerto Rico, Rio Piedras Campus, San Juan, Puerto Rico, United States of America; 2 Department of Biology, Functional Genomics Research Core, University of Puerto Rico, Rio Piedras Campus, San Juan, Puerto Rico, United States of America; 3 Department of Biology, University of Puerto Rico, Rio Piedras Campus, San Juan, Puerto Rico, United States of America; 4 Department of Chemistry, University of Puerto Rico, Rio Piedras Campus, San Juan, Puerto Rico, United States of America; 5 High Performance Computing Facility, University of Puerto Rico, Central Administration, San Juan, Puerto Rico, United States of America; Universidade de São Paulo, Brazil

## Abstract

Voluntary running is a robust inducer of adult hippocampal neurogenesis. Given that fatty acid synthase (FASN), the key enzyme for *de novo* fatty acid biosynthesis, is critically involved in proliferation of embryonic and adult neural stem cells, we hypothesized that FASN could mediate both exercise-induced cell proliferation in the subgranular zone (SGZ) of the dentate gyrus (DG) and enhancement of spatial learning and memory. In 20 week-old male mice, voluntary running-induced hippocampal-specific upregulation of FASN was accompanied also by hippocampal-specific accumulation of palmitate and stearate saturated fatty acids. In experiments addressing the functional role of FASN in our experimental model, chronic intracerebroventricular (i.c.v.) microinfusions of C75, an irreversible FASN inhibitor, and significantly impaired exercise-mediated improvements in spatial learning and memory in the Barnes maze. Unlike the vehicle-injected mice, the C75 group adopted a non-spatial serial escape strategy and displayed delayed escape latencies during acquisition and memory tests. Furthermore, pharmacologic blockade of FASN function with C75 resulted in a significant reduction, compared to vehicle treated controls, of the number of proliferative cells in the DG of running mice as measured by immunoreactive to Ki-67 in the SGZ. Taken together, our data suggest that FASN plays an important role in exercise-mediated cognitive enhancement, which might be associated to its role in modulating exercise-induced stimulation of neurogenesis.

## Introduction

Numerous human and animal studies have clearly demonstrated that voluntary exercise enhances and protects diverse aspects of brain function. Human studies report that running improves learning and memory, as well as executive function, while counteracting mental decline [1], [2], [3], [4], [5]. In rodents, running has positive effects on dentate gyrus (DG) neurogenesis [6], long-term potentiation [6], growth factors and neurotrophins, including brain-derived neurotrophic factor [5], [7], stress reduction [8], cerebellar and motor cortex angiogenesis [9], [10], and reduction of oxidative protein modification [11]. Moreover, research on rodents demonstrates that running improves hippocampal-dependent learning and memory [7], [11], [12], [13], [14], [15], [16], [17].

While the cognitive and memory enhancing effects of exercise at the physiological and molecular levels have been intensively investigated, the underlying biochemical mechanisms, have received less or no attention. Of interest to us was the idea that relevant metabolic perturbations, as well as specific factors related to lipid metabolism could be associated with the beneficial effects of exercise on hippocampal-dependent learning and memory and neurogenesis. Lipids account for about 50% of the brain’s dry weight [18]. In neurons, fatty acids (FA) are esterified predominantly into phospholipids that stimulate membrane production. Bioactive FA modulate protein function directly by posttranslational modification, while also acting as important second messengers [19]. FA may function as intracrine messengers or as paracrine neuromodulators, possibly contributing to the maintenance of neuronal networks associated with learning and memory. Interestingly, various mental illnesses characterized by improper cognition, including manic depression and schizophrenia, as well as neurodegenerative disorders such as Alzheimer’s, Parkinson’s and Niemann-Pick diseases, are associated with impaired lipid metabolism [20], [21].

Saturated FA are derived from dietary origin (exogenous), yet they also may be produced endogenously *de novo* via biosynthesis by FA synthase (FASN). FASN is a homodimer with two identical multifunctional polypeptides, each including seven catalytic domains: beta-ketoacyl synthase, malonyl/acetyltransferase, dehydrase, enoyl reductase, beta-ketoacyl reductase, acyl carrier protein, and thioesterase [22]. Palmitate (PA), a saturated 16-carbon FA, is the predominant product of FASN synthesized from the three primer substrates, acetyl-CoA, malonyl-CoA, and NADPH, can also be elongated by FASN to stearate (SA) [23]. Expression, and thus function and activity, of FASN is regulated principally at the transcriptional level via signaling mechanisms coupled to activation of transcriptional factors such as, upstream stimulatory factors (USFs), sterol regulatory element binding protein-1 (SREBP-1) [24], and Peroxisome Proliferator-activated receptor-gamma (PPARγ) nuclear receptors [25] In addition, the biosynthesis of FA can be reduced by Spot14, which decreases the availability of malonyl-CoA, one of the primer substrates for FASN [26].

Since FASN is critically involved in proliferation of adult neural stem cells [26], we hypothesized that mice experiencing voluntary exercise versus sedentariness, would differ in FASN expression in the hippocampus and that this effect could be associated with improved cognition coupled to enhanced DG cellular proliferation. Results demonstrated that voluntary exercise specifically induces hippocampal upregulation and accumulation of *fasn* mRNA as well as that of PA and SA. Moreover, blocking exercise-induced forebrain activity of FASN with its inhibitor, C75, disrupted exercise-mediated cognitive enhancement tested in the hippocampal-dependent spatial Barnes maze and impaired the proliferative response, also induced by exercise, in the subgranular zone (SGZ) of the DG. Overall, we have identified a lipid biosynthetic machinery involving FASN as a mediator of important exercise-induced benefits in 20 week-old male mice on hippocampus-dependent spatial learning and memory and cellular proliferation in the SGZ of the DG.

## Experimental Procedures

### Animals

Seventy-three 20 week-old male C57BL/6J mice (The Jackson Laboratory) were divided into six groups: Sedentary (n = 12), Running (n = 12), vehicle-Sedentary (VHL-S, n = 10), vehicle-Running (VHL-R, n = 10), sham-Sedentary (SH-S, n = 5), sham-Running (SH-R, n = 5), C75-Sedentary (C75-S, n = 9), and C75-Running (C75-R, n = 10). Mice were housed individually in temperature-controlled (21°C) quarters with a 12-h light/12-h dark cycle and were provided with water and food (Purina Chow) *ad libitum*. All protocols were approved by the Institutional Animal Care and Use Committee of the Río Piedras Campus at the University of Puerto Rico in compliance with the guidelines of the National Institutes of Health (NIH) for the care and use of laboratory animals (Department of Health and Human Services–NIH publication number 86-23).

### Voluntary Exercise Paradigm

**Figure 1:**
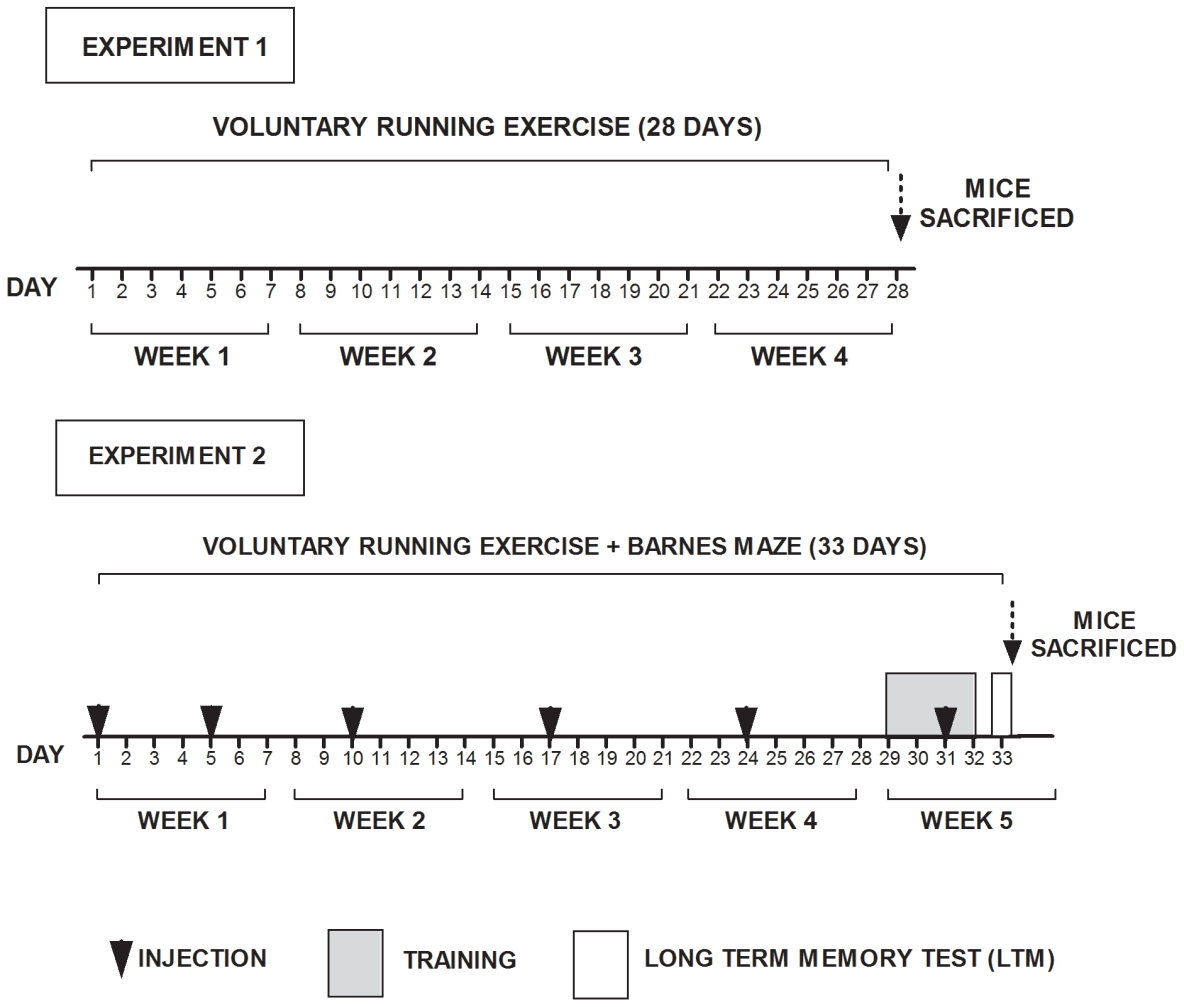
Experimental design. **Experiment 1.** Mice were allowed to run for 28 days and were then sacrificed followed by the analysis of FA content in the brain and of the expression of genes encoding metabolic enzymes. **Experiment 2**. Mice were allowed to run for 33 days and given chronic i.c.v. injections of C75 (▾) at the indicated times. On day 29^th^ of running, Barnes maze behavioral testing was initiated, which consisted of five days. The complete running period spanned the entire experimental design for 33 days.

Sedentary controls were housed individually for one month in a standard cage without any equipment to exercise. The experimental physically active groups were housed under the same conditions as their sedentary counterparts with the only exception that they had continued access to an unlocked wireless low profile running wheel (Med Associates), located within their home cages. The experimental design (**Fig. 1**) included two independent experiments and groups of mice. In **Experiment 1**, mice exercised for 28 days, after which they were immediately sacrificed and their brains used for the analysis of FA and the expression of genes encoding metabolic enzymes (see description below). In **Experiment 2,** mice exercised for an entire period of 33 days and received chronic intracerebroventrically (i.c.v.) injections of C75 at the indicated times (**Fig. 1**). On the 29^th^ day of running, we began to administer the Barnes maze behavioral test (see description below), which was given daily for 5 d. After completion of the Barnes maze behavioral testing, mice were sacrificed immediately followed by the carrying out of histological procedures (see description below).

### Stereotaxic Brain Surgery

Mice were handled for 2–3 d before undergoing surgery as described by us previously [27]. For surgery, mice were anesthetized with 1.25% tribromoethanol (0.025 ml/g body weight, i.p.), and placed into a stereotaxic apparatus (David Kopf Instruments), with the nose angled at 0°. Bilateral guide cannulae (6 mm long) were implanted above the lateral ventricles using the following coordinates: anterior–posterior, −0.6 mm from bregma; medio-lateral, ±1.2 mm from midline; dorso-ventral, 1 mm from skull located following the stereotaxic coordinates form the adult mouse brain atlas [28]. The cannulae were secured to stainless-steel screws with dental cement. Wire stylets were inserted into the cannula guides and checked every day to ensure clean and functional cannulae. After surgery, animals were allowed to recover for 4 d before behavioral experiments.

### Intracerebroventricular Microinfusions of the FASN Inhibitor C75

Trans-4-carboxy-5-octyl-3-methylenebutyrolactone (C75, Sigma-Aldrich) was dissolved in sterile dimethyl sulfoxide [DMSO] (Sigma-Aldrich) at a concentration of 100 µg/µl [29] and diluted to 10 µg/µl using 70% ethanol. I.c.v. microinfusions were administered through two stainless steel injectors (7.2 mm) inserted into cannulae. The total volume of 1 µl was infused at a constant rate of 0.5 µl min^–1^ attached to a 10-µl Hamilton micro-syringe. Infusions were administered along the 33 d running or sedentary periods, as indicated in **Fig. 1**. Running and Sedentary mice received 10 µg C75 i.c.v. or VHL (10% DMSO in 70% ethanol), respectively, on Days 1 and 5 of **Experiment 2** followed by additional infusions on Days 10, 17, 24 and 31 (**Fig. 1**). The cohort of VHL control mice was pair-fed and yoked to the C75-treated animals. The amount of food consumed by C75-treated mice was determined by weighing the food daily (grams). Pair-fed, yoked control animals, received the amount of food consumed by their C75-treated counterparts immediately after injection. Animal running activity and weights were recorded weekly.

### Assessment of Hippocampal Dependent Memory using the Barnes Maze

The Barnes maze is a behavioral task that assesses hippocampal-dependent spatial learning and memory in rodents [30]. In our study, we used the Barnes Maze test because it is more ethologically relevant, less stressful, and has a minor physical activity component compared to the Morris Water Maze when it comes the examination of spatial learning in rodents. The maze used here is a circular platform with 16 equally spaced holes along the perimeter and is elevated 105 cm above the floor. All holes look identical from most points on the maze. Two reinforcements, bright light and a blowing fan, were used as aversive stimuli to motivate mice to perform the task of successfully searching for the escape hole. In addition, two extra-maze visual cues with different colors and shapes, a dark grey cartoon with white stripes and a round black fan, were placed at two sides of the maze. A Gigaware®25157 webcam was mounted to the ceiling focusing on the maze surface to record all trials for analysis of behavioral parameters. The experimenters conducting the analysis were blind to the types of treatment of all mice. The task was performed as described before [31] and included an adaptation period, a spatial acquisition period that included one session per day given for four consecutive days; each session consisting of four 3 min trials with 15 min inter-trial intervals. The long-term memory (LTM) test was performed on day 5 and consisted of 1 session exactly as described above. The behavioral parameters evaluated included: (a) latency to enter into the escape hole - time from the start of the trial to the first entry into the target hole (min); (b) error frequency in the LTM test - number of visits to holes other than the target hole; and (c) search strategy - random search strategy, characterized by a non-systematic exploration of the maze with many center maze crossings; serial search strategy, defined as systematic, thigmotaxic, consecutive hole searching in a clockwise or counter clockwise manner; and spatial search strategy, described as searching less than three holes from the location of the goal [32]. A cued escape hole control test was not used because contrary to the Morris water maze, this type of control cannot be used to dissociate between spatial and non spatial learning in the Barnes maze [33]. In addition, assessment of motor capacity within groups was assessed during the adaptation exploratory phase upon introduction of mice to the Barnes maze.

### Brain Tissue Preparation

After completion of the 28 d period of sedentariness or voluntary running (**Fig. 1** **, Experiment 1**), mice were killed by decapitation, blood was collected from their body trunk, and their brains were rapidly extracted from the cranial vault, chilled in ice-cold Phosphate Buffer Saline (PBS, Sigma-Aldrich), followed by placing them on dry ice. The hippocampal and cortical tissue (cortical layers I through VI anterior-posterior and medial-lateral) samples were frozen immediately in liquid nitrogen after dissection. Next, the frozen tissues were weighed in order to ensure that we reached the required 100 mg tissue mass needed for FA extraction. For each condition, six hippocampi and cortical tissue pools (each consisting of 100 mg of bilateral tissue pooled from pairs of animals per condition) were obtained from a total of 12 animals and stored at −80°C. Cerebella were obtained from one animal per condition, for a total of 6 animals used per condition, and 100 mg of tissue was processed for FA extraction as above. Blood samples were pooled from two animals per condition to achieve 1 mL samples that were immediately frozen at −80°C for subsequent FA extraction. Finally, 100 mg of a diet specimen was also used for FA extraction.

### FA Extraction, Etherification and Analysis

Brain tissue samples were homogenized with 2 mL chlorophorm/methanol (CHCl_3_/MeOH) (1∶1) (Sigma-Aldrich) on ice using a modification of the method of Bligh and Dyer [34]. The samples were vortexed and remained on ice for 15 min, followed by centrifugation at 10,000 × g for 5 min at 4°C. The resulting CHCl_3_ phase was evaporated to dryness under vacuum and the samples were transmethylated with MeOH and HCl (Sigma-Aldrich) for 24 h at −20°C. FA methyl esters were extracted in ether, cleansed by filtering through silica gel columns, evaporated, and dissolved in 5 µl hexane for further analysis. FA profiling was performed using an Agilent 7890A series GC/MS (Agilent Technologies) equipped with a 30 m × 0.25 mm special performance capillary column (HP-5MS) of polymethyl siloxane cross-linked with 5% phenyl methylpolysiloxane. The obtained mass spectra were interpreted by comparison of retention times with a group of internal FA methyl esters’ standards (Sigma-Aldrich) previously separated on the same gas chromatograph using the Agilent 5975C MS ChemStation software [35] and by cross-checking with those registered in databases. Quantitation of each FA was carried out using each peak’s height area, which was divided by the sum of all the peaks in the FA profile for final expression as a ratio of the total of all FA peaks.

### RNA Extraction, Reverse Transcription and Real time PCR

Hippocampi, cortex and cerebella were dissected as described in the tissue preparation section for FA extraction, placed in RNA stabilization reagent (RNAlater; QIAGEN), and stored at −80°C. RNA extraction was done using an RNA isolation kit (QIAGEN) according to the manufacturer's instructions and as previously described by us [36]. The concentration and integrity of RNA were determined using the NanoDrop-1000 Spectrophotometer (NanoDrop Technologies). cDNA was obtained from 1 µg of total RNA samples using the TaqMan reverse transcription (RT) reagents (Applied Biosystems) as described previously by us [36].

For Real time PCR analysis, primer sequences obtained from Primer Bank [37] were as follows: *fasn* (ID 30911099a2); *gapdh* (ID 6679939a1). Previously published primer sequences were used to analyze *elovl 1* and *elovl 6* mRNA changes [38]. All primers were synthesized by Sigma-Aldrich. Real-time PCR was performed using an iCycler iQ Real time PCR Detection System (Bio-Rad) and the QuantiTect SYBR Green PCR kit (QIAGEN). Briefly, cDNA diluted 10 times with sterile water was combined with 0.1 µM of each primer, 1× QuantiTect SYBR Green PCR Master Mix (HotStarTaq DNA polymerase, quantiTect SYBR Green PCR buffer, dNTP mix, and SYBR Green I), and PCR-grade water to a volume of 20 µl. The cycling conditions for all primers were as follows: 95°C for 15 min to activate the HotStarTaq polymerase, followed by 40 cycles consisting of three steps, 45 s at 95°C (denaturation), 30 s at 60°C (annealing), and 30 s at 72°C (extension). The PCR program was completed by a melting temperature analysis consisting of 1 min at 95°C (denaturation), 2 min at 55°C (annealing), and then 101 steps lasting 8 s each, through which temperature ranged from 55 to 95°C.

To analyze the change in the expression of target genes *fasn*, elongases 1 and 6 (*elovl1/6*) relative to the reference gene *gapdh*, we used a java desktop application DeltaDeltaCT_v02 (NetBeans IDE) developed at the High Performance Computing facility at the University of Puerto Rico. The program implements the delta-delta Ct method of qRT PCR [39] including a verification of its applicability. The application has a convenient user-friendly interface to enter Ct values from the Real-Time PCR data as well as means to view and save results in tables, line graphs and diagrams. Using paired and unpaired statistics, users can evaluate the relative expression of a set of different genes or the dynamics of a pair of genes. The program is freely available upon request (analtoliy.chornyy@upr.edu).

### Immunohistochemistry

All groups of animals that had received C75 or VHL infusions including sham controls were sacrificed immediately after the end of the memory test (**Fig. 1** **Experiment 2**). Brains were dissected, washed with chilled PBS, and stored at −80°C. Fresh frozen alternate coronal sections (20 µm thick) obtained in a cryostat at −20°C, were placed on positively charged glass slides (Fisher Scientific) and stored at −80°C. Sections were then fixed with 2% paraformaldehyde, permeabilized with 0.2% Triton X-100 in 0.1% of sodium citrate, and washed with PBS. Antigen retrieval was performed by sequential microwaving (Panasonic, NN4425A) for 105 s at power 10 and then for 10 min at power 1 in 0.01 M citrate buffer. Next, the sections were incubated with 0.3% H_2_O_2_ in PBS for 30 min, washed again with PBS, and incubated with 5% goat serum in PBS for 30 min. The primary anti-Ki-67 antibody (EMD Millipore Headquarters) was diluted at 1∶100 in blocking solution (1% goat serum in PBS) and incubated overnight at 4°C followed by washing with PBS. Next, slides were incubated for two hours with a secondary biotinylated affinity-purified goat anti-rabbit IgG diluted at 1∶100 followed by washing with PBS. Detection of primary immunoreactivity was achieved with the Ultra-Sensitive ABC Peroxidase Rabbit IgG Staining Kit (Thermofisher Scientific), used according to the protocol detailed by the manufacturer. The color was developed with 0.04% H_2_O_2_ and 0.05% DAB. Omission of the primary antibody was used as a negative immunohistochemical control and resulted in no Ki-67 staining (data not shown). The slides were mounted using permanent mounting medium (Vector Laboratories). Images were captured by Olympus BX40 microscope equipped with a Polaroid DMC digital camera (1600×1200 dpi) with 12× objective magnification. Images were quantified using the Image J program (http://rsb.info.nih.gov/ij/).

### Statistics

Statistical analysis was performed with the Prism software (GraphPad, La Jolla, CA). Statistical significance was assumed at p<0.05 and determined by One-Way ANOVA or Two-Way repeated measures ANOVA (RM ANOVA) followed by Bonferroni tests for multigroup comparisons. Bioinformatics analysis of obtained FA spectra was performed using web-based tools for quantitative metabolomics: Metaboanalyst [40]. The intrinsic variations within FA data sets in each brain region of sedentary and running mice were examined by Principal Component Analysis (PCA). In PCA component loadings are simple correlations (using Pearson’s *r*) between the components and the original variables. Loading values above zero are considered to be positive loadings and reflect a high degree of positive contribution in favor of the components displaying such positive values.

## Results

### Running-induced Hippocampal Specific Upregulation of *fasn* mRNA

**Figure 2:**
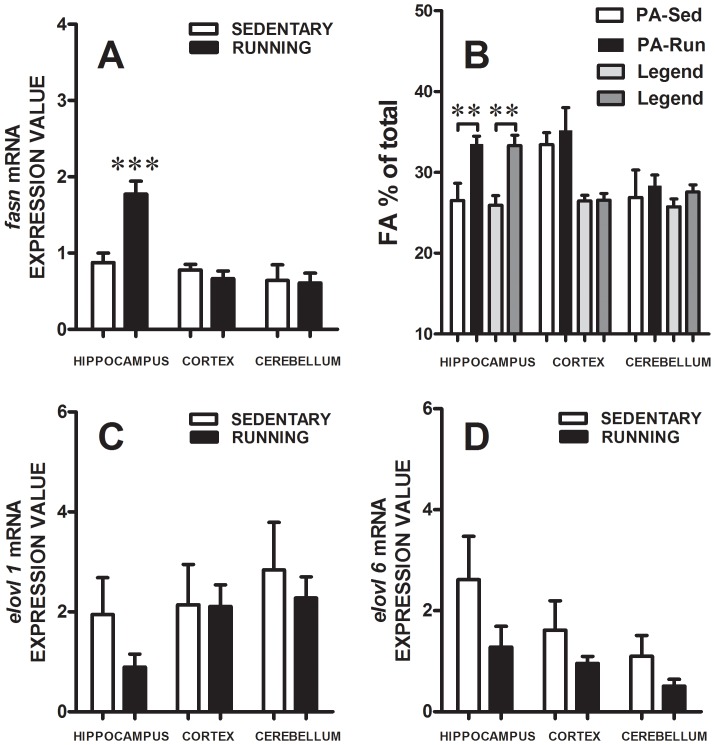
Voluntary running increases *fasn* gene expression and the content of PA and SA in the hippocampus, but not in cortex, or cerebellum. (A) Increase in *fasn* gene expression by running. Amplification plots were produced to calculate the threshold cycle (Ct) followed by evaluation of the changes in the expression of target *fasn* relative to our reference gene, *gapdh,* using our java desktop application DeltaDeltaCT_v02 (see Materials and Methods). Data are expressed as the mean value using the estimated 95% Ct, represented as the mean ± SEM. Bonferroni post-testing identified a specific significant increase in hippocampal *fasn* mRNA levels in running versus sedentary mice (***P<0.001). Sed - sedentary, Run - running. (B) Running-dependent elevation of PA and SA in the hippocampus. Quantitation of PA and SA was carried out using each peak’s height area and the sum of all peaks from the GC/MS data. Results were expressed as a percent of total of all peaks (mean ± SEM), N = 6. Data were analyzed with Two-Way RM ANOVA followed by Bonferroni post-testing; **P<0.01 indicates specific statistically significant differences, as compared with corresponding Sedentary controls. (C, D) The expression of *elovl1* and *elovl6* in the brain is not potentiated by running. Amplification plots were produced to calculate the threshold cycle (Ct) followed by evaluation of the changes in the expression of target *elovl 1* and *elovl 6*, respectively, relative to our reference gene, *gapdh,* using our java desktop application DeltaDeltaCT_v02 (see Materials and Methods). Data are expressed as the mean value using the estimated 95% Ct, represented as the mean ± SEM.

Mice exposed to the running wheel ran an average distance of 7.19±1.3 km/day. All running mice experienced a 6% loss of body weight during week 1. After 28 days, running mice lost 13±3%, while sedentary mice gained 8±1%, of body weight (Two-Way RM ANOVA: Exercise - F_(1,72)_ = 55.62, ***P<0.0001; Running Days - F_(4,72)_ = 9.138, ***P<0.0001; Interaction - F_(4,72)_ = 19.09, ***P<0.0001). Post-testing analysis demonstrated that during the first two weeks of experiment both groups displayed similar weight (*P*<0.05), while significant weight loss was observed in running group through Days 21–28 (***P<0.001). Importantly, as seen in **Fig. 2** **,A**, we identified a dramatic and brain region-specific up-regulation of *fasn* mRNA in running mice (Two-Way RM ANOVA: Region - F_(2,16)_ = 15.05, ***P<0.0005; Exercise - F_(1,16)_ = 4.653, *P*>0.05; Interaction - F_(2,16)_ = 8.267, **P<0.005). Bonferroni post-testing indicated that such induction of *fasn* mRNA by running was specific to the hippocampus (***P<0.001), not being observed in the cortex or the cerebellum (*P*>0.05, each).

Next, we aimed to evaluate whether this increase in *fasn* gene expression could be accompanied by hippocampal-specific changes in FA metabolism as opposed to it constituting an adaptive response to increased food intake triggered by voluntary running. Thus, we analyzed FA content in blood, hippocampus, cortex, and cerebellum of both Running and Sedentary groups by GC/MS. No changes in the content of FA in response to running were detected in blood. FA displaying the highest abundances in blood were PA>α-linoleate (C18∶2, ω6) = SA>oleate>arachidonate>docosahexaenoic acid (data not shown). Among the selected brain regions we detected and profiled twenty FA. It is noteworthy that for our continued analysis in the study we used only FA with an even number of carbons, representing those that can be derived from both endogenous and exogenous sources. FA having an uneven number of carbons and thus, known to have only exogenous sources were excluded from our analysis: C15∶0, C17∶0, C19∶0, C23∶0. As seen in **Table 1**, the FA displaying the highest abundances in the brain were PA (C16∶0) = SA (C18∶0)>oleate (C18∶1, ω9)>arachidonate (C20∶4, ω6) ≤ docosahexaenoic acid (C22∶6, ω3). As determined by Two-Way RM ANOVA and as seen in **Fig. 2** **,B**, PA and SA were significantly elevated by running (Exercise: F_(3,45)_ = 8.518, ***P<0.0001; Region: F_(2,45)_ = 2.497, *P*>0.05; Interaction: (F_(6, 45)_ = 4.187, **P<0.01; Subject Matching: F_(15, 45)_ = 2.114, *P<0.05). Post-testing analysis showed that running animals had higher levels of both PA and SA than sedentary mice in the hippocampus (**P<0.01), but not cortex or cerebellum (*P*>0.05 each). This running-induced accumulation of hippocampal PA and SA may be related to the running-induced enhancement of *fasn* gene expression (see **Fig. 2** **,A**), ensuing in increased FASN activity and elevated FA biosynthesis.

**Table 1:** FA content in the brain regions of Sedentary (S) and Running (R) C57BL/6J male mice.

FA	HS	HR	CXS	CXR	CRS	CRR
**C14∶0**	0.3±0.1	0.1±0.1	0.1±0.1	35.2±2.9	0.1±0.1	0.1±0.1
**C16∶0**	26.5±2.1	32.5±1.0	33.4±1.5	26.6±0.8	26.9±3.4	28.3±1.4
**C18∶0**	25.9±1.2	33.3±1.3	26.5±0.7	0.2±0.1	25.7±1.0	27.6±0.9
**C20∶0**	0.4±0.1	0.1±0.05	0.1±0.1	0.2±0.1	1.6±0.6	1.3±0.8
**C22∶0**	0.4±0.1	0.2±0.1	0.1±0.1	0.1±0.1	0.5±0.2	0.2±0.1
**C24∶0**	0.4±0.1	0.2±0.1	0.4±0.2	0.1±0.1	0.6±0.1	0.5±0.3
**C16∶1**	0.5±0.04	0.4±0.1	0.6±0.1	16.6±0.4	0.5±0.1	0.4±0.1
**C18∶1**	19.9±0.6	16.3±2.6	17.7±1.3	0.6±0.1	20.7±1.6	25.4±1.1
**C20∶1**	1.8±0.2	0.8±0.1	0.9±0.3	0.1±0.1	3.8±1.2	2.8±0.7
**C22∶1**	0.2±0.04	0.1±0.1	0.1±0.1	0.3±0.1	0.2±0.1	0.1±0.1
**C24∶1**	1.1±0.4	0.3±0.1	0.3±0.1	0.3±0.1	1.0±0.3	0.7±0.2
**C20∶2**	0.2±0.03	0.1±0.1	0.2±0.2	0.3±0.1	0.3±0.1	0.2±0.1
**C20∶3**	0.4±0.1	0.2±0.1	0.3±0.1	7.3±1.6	2.1±1.4	1.5±1.3
**C20∶4**	9.3±0.8	6.8±0.8	8.2±1.1	1.9±0.8	5.8±1.6	4.4±0.8
**C22∶4**	2.6±0.5	1.3±0.2	1.5±0.2	1.9±0.8	2.3±0.6	1.3±0.4
**C22∶6**	10.2±1.5	6.3±0.8	9.5±1.5	9.9±1.5	7.8±0.8	5.1±0.7

Data are expressed as the percent of the total methylated FA ± SEM, N = 6. HS - hippocampus sedentary; HR- hippocampus running; CXS – cortex sedentary; CXR – cortex running; CRS – cerebellum sedentary; CRR – cerebellum running.

Since, SA can be produced not only by FASN, but also by ELOVL1 or ELOVL6 via chain elongation of PA, we examined the expression levels of *elovl1* and *elovl6* in tested brain regions of running and sedentary mice. Interestingly, as seen in **Figs. 2** **,C,D**, we found no expression changes in *elovl1* (Two-Way RM ANOVA: Region - F_(2,16)_ = 1.228, *P*>0.05; Exercise - F_(1,16)_ = 2.598, *P*>0.05) or *elovl6* mRNAs (Two-Way RM ANOVA: Region, F_(2,16)_ = 2.837, *P*>0.05; Exercise, F_(1,16)_ = 4.277, *P*>0.05). These results demonstrate that of the three enzymes, only FASN is upregulated in the hippocampus as a result voluntary exercise. Thus, FASN, and not ELOVL1/6, is possibly responsible for the hippocampal-specific increases in both PA and SA induced by running; SA through *de-novo* chain elongation from PA [23].

### PCA of the FA Spectra Obtained by GC/MS

**Figure 3:**
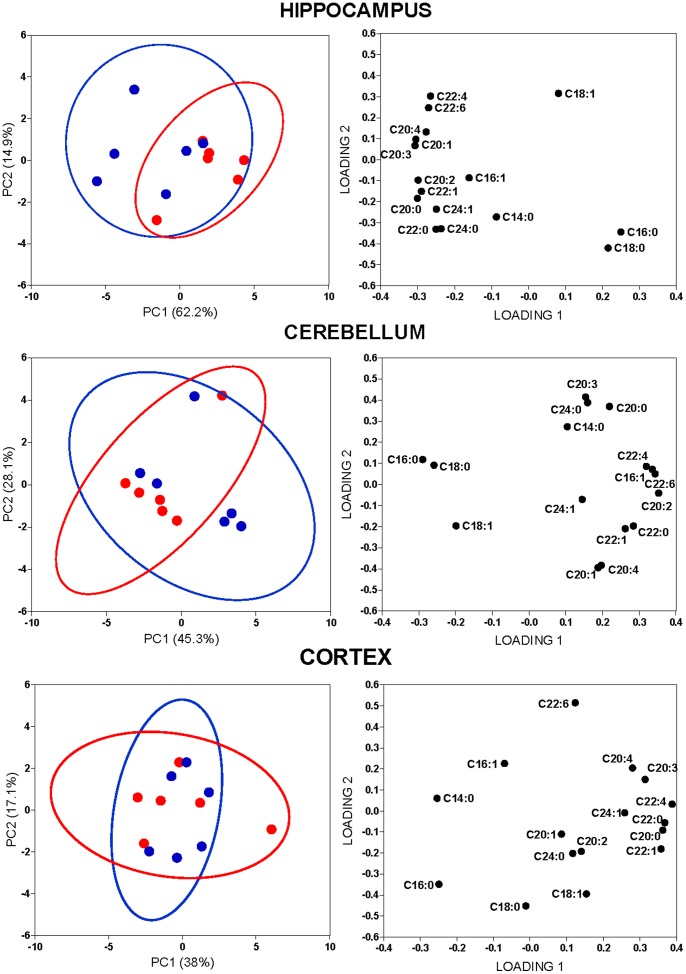
Identification of FA that influence the separation between Running and Sedentary groups in the neurolipidomics analysis. Ellipses of the score plots (**Left**) generated after PCA analysis of variations in FA data sets (N = 6 per brain region) including corresponding loading plots (**Right**), in the hippocampus, cerebellum, and cortex of sedentary (blue circle) or running (red circle) mice. The ellipses of the score plots illustrate 95% confidence region of the groups.

PCA was used to identify other FAs that may be essential for identification of metabolic differences between sedentary and running animals according to the brain regions examined (**Fig. 3**). In the hippocampus, the combined explained variance of PC1 and PC2 was equal to 78.6% displaying a distinguished separation of the two groups mainly along the direction of PC1, which carried 63.8% of the total variance of the FA data **(Top Left).** In our analyses, assessment of the PC loadings indicated that the positive contribution for variance of PC1 between sedentary and running mice was made by the contribution of two specific saturated FA: PA (C16∶0) and SA (C18∶0), while a negative contribution was made by several FA: C20∶0, C22∶0, C20∶4, C20∶1, C22∶1, C24∶1, C20∶2, C20∶3, C22∶4 and C22∶6 (**Top Right**). These data served as further confirmation that among all the FA identified, only PA and SA increased in the hippocampus of running animals, but not in sedentary controls.

In contrast, the pattern of separation between groups in the cerebellum was less clear. Despite of the fact that the first two components, PC1 and PC2, captured 75.9% of the total variance in the FA data from the cerebellum, there was no crucial dominance of PC1 (47.5%) over PC2 (28.4%) (**Mid Left**). Inspection of the PC loadings indicated that the FA contributing positively to PC1 were C22∶0, C16∶1, C22∶1, C20∶2, C22∶4 and C22∶6, whereas C16∶0 and C18∶0 made a negative input (**Mid Right**), contrary to our findings in the hippocampus. The FA contributing to PC2 were C14∶0, C20∶0, C20∶4, C20∶3 and C22∶0, C20∶1, C22;1 and C20∶4, correspondently. PCA analysis of the cortex did not reveal any notable differences between sedentary and running mice, as seen also in the cerebellum. In the cortex, two major principal components, PC1 and PC2, carried 58.2% of the total explained variance of the FA data sets (**Bottom Left**). PC loading identified that C20∶0, C22∶0, C22∶1, C24∶1, C20∶3 and C20∶4 contributed positively to the PC1 components in the cortex. Alternatively, the PC1 loading identified C14∶0, C16∶0 and C18∶0 as negative contributors to the PC components in the cortex. The FA contributing positively to PC2 were C14∶0, C16∶1, C20∶4 and C22∶6, while those contributing negatively were C18∶0, C18∶1 and C20∶1 (**Bottom Right**).

Thus, we found that FA species that influenced PC loadings were different between brain regions. For example, in the hippocampus, PC1 had a high positive loading from only two saturated FA, namely C16∶0 (PA) and C18∶0 (SA), while in the cerebellum and cortex the contribution of the same lipid species was negative. Thus, PCA analyses suggest that among the studied brain regions, running specifically elevates the abundance of PA and SA in the hippocampus, a brain region in which exercise exerts multiple cellular effects including the observed upregulation of *fasn* reported here (**Fig. 2** **,A**).

### Functional Roles of FASN in the Hippocampus

Based on our results so far, we hypothesized that if FASN expression and activity are potentiated by exercise, the molecule could play an important role in running-induced changes in hippocampal-dependent processes, such as spatial learning and memory. Thus, we next utilized i.c.v. infusions of C75, a specific, competitive, irreversible inhibitor of FASN resembling malonyl-CoA, one the priming substrates of the enzyme [41], [42]. C75 causes profound, dose-dependent, anorexia that lasts only three hours [43]. Thus, in our experimental design, to avoid the reported resistance of lean mice to C75 [44], we subjected our subjects to two C75 or vehicle (VHL) i.c.v. injections during the first week of the study, which were followed by single weekly i.c.v. injections of the drug during next four weeks (see **Fig. 1** **, Experiment 2**). We chose to administer C75 i.c.v., instead of intrahippocampally, in order to target the entire forebrain. The molecular and biochemical changes observed were seen in the hippocampus and not in the cortex or the cerebellum. However, our results did not discern in what hippocampal subregion did these changes occur and did not rule out subcortical regions or brain areas outside of layers I to IV of the cortex. Thus, in order to increase the possibility that we would observed any effects by C75, injections were given i.c.v., rather than intrahippocampally in order to reduce the possibility of a false negative result.

In our study, C75 treatment reduced food intake in mice to 4.6±0.3 g of chow diet/day, similar to results reported previously [45]. In order to control for these reported C75 anorexigenic effects, we pair-fed the VHL injected Sedentary (VHL-S), VHL injected Running (VHL-R), sham Sedentary (SH-S) and sham Running (SH-R) mice, yoking them to the amount of food ingested by their C75 treated counterparts, (C75-S) and (C75-R). Our results found no significant differences in body weight between groups after i.c.v. injections (data not shown). In addition, C75 infusions did not alter running performance of treated animals compared to controls (Two-Way RM ANOVA: Treatment - F_(1,68)_ = 0.004206, *P*>0.05; Running Days - F_(4, 68)_ = 17.26, ***P<0.0001), agreeing with a previous report indicating that C75 did not affect generalized locomotor activity [46] and suggesting that C75 had no general physiological or motor toxicity. Indeed, we did not observe any evidence of central or peripheral neurotoxicity resulting in diarrhea, excessive urination, physical discomfort or aggressive behavior in treated animals. These observations are consonant with those previously published [47].

### C75, Running, and Escape Latency in the Barnes Maze

**Figure 4:**
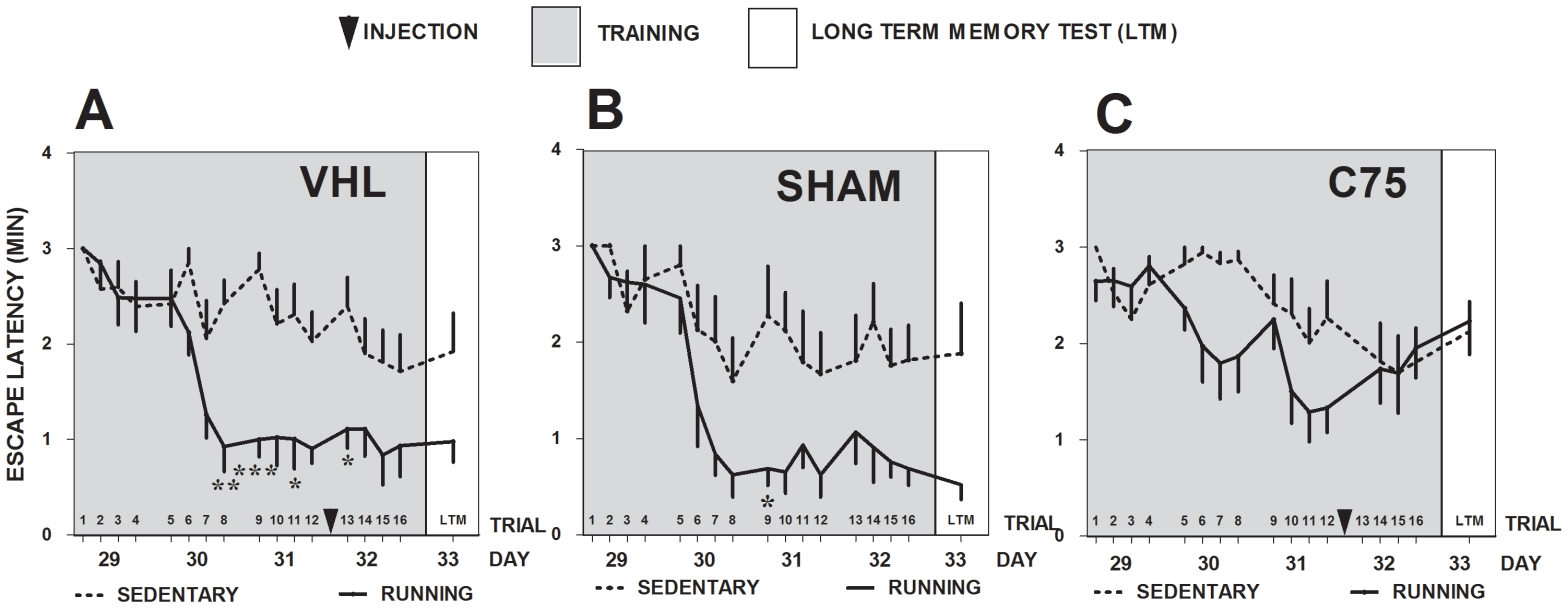
Effects of C75 on searching strategy during Barnes maze acquisition and LTM testing. Acquisition and LTM data depicting the particular searching strategies, random (black circle), serial (empty circle), or spatial (red circle), used to search for the escape hole within the Barnes maze as observed for treated and control mice of both Sedentary and Running groups VHL-S (**A**), VHL-R (**B**), SH-S (**C**), SH-R (**D**), C75-S (**E**) and C75-R (**F**). Graphed data represent the % of mice per session (consisting of 4 trials per day) in each group using selected searching strategies throughout acquisition and LTM testing. Two way RM ANOVA and Bonferroni post-testing (*P<0.05, **P<0.01, ***P<0.001) indicates specific statistically significant differences between groups in terms of the searching strategies preferred for searching (*-spatial vs. random; **^#^**-spatial vs. serial, **^&^**- serial vs. random).

We next directly assessed whether blocking running-induced FASN function with i.c.v. C75 would affect the beneficial effects of exercise on spatial memory in the Barnes maze. Following the four weeks of running and corresponding infusions for all the groups used in our studies (VHL-S, VHL-R, SH-S, SH-R, C75-S and C75-R), mice from each group were subjected to four days of Barnes Maze acquisition training and an additional day for LTM testing 24 h after acquisition. Barnes maze training was conducted between Days 29 and 32 of the experimental design, while LTM was tested on Day 33 (**Fig. 1** **, Experiment 2**). To begin to assess the results of the experiment, we calculated the mean escapes latencies of each animal according to trial performances during acquisition and LTM testing. The data was then subjected to Two-Way RM ANOVA followed by Bonferroni post-testing. VHL-R mice (**Fig. 4** **,A**) displayed significantly shorter escape latencies than VHL-S controls, even though both groups did display significantly reduced latencies as training trials increased, indicating that, while Running mice performed better in the Barnes maze than the Sedentary controls, animals in both groups learned during the task (Running Factor: F_(1, 288)_ = 9.888, ***P<0.0001; Trials Factor F_(16,288)_ = 10.89, ***P<0.0001; Interaction: F_(16, 288)_ = 3.502, **P<0.005; Subject Matching: (F_(18, 288)_ = 9.234, ***P<0.0001). Post-testing analysis demonstrated that the VHL-S group was significantly impaired in LTM compared to the VHL-R group on Days 30 - trial 8 (**P<0.01), 31 - trial 9 (***P<0.001), 31– trial 11 (*P<0.05) and 32– trial 13 (*P<0.05). These results reiterate the multiple findings by others, establishing that running improves spatial learning [7], [11], [12], [13], [14], [15], [16], [17]. Similar results were obtained when comparing SH-R versus SH-S groups (**Fig. 4** **,B**). SH-R mice displayed significantly reduced escape latencies compared to SH-S controls during acquisition training, while both groups did display significantly reduced latencies as the number of training trials increased, also indicating that Running and Sedentary mice learned during the task (Two-Way RM ANOVA: Running Factor - *F*(_1,128_) = 24.91, **P<0.005; Trials Factor - F_(16,128)_ = 7.930, ***P<0.0001; Interaction - F_(16, 128)_ = 1.382, *P*>0.05; Subject Matching - F_(8, 128)_ = 2.061, *P<0.05). Post-testing analysis demonstrated that mice in the SH-S group were significantly impaired compared to those in the SH-R group on Day 31 - trial 9 (*P<0.05). Interestingly, as observed in the plotted data for both VHL-R (**Fig. 4** **,A**) and SH-R (**Fig. 4** **,B**) groups, a dramatic decrease in escape latency and thus a high expression of learning activity was observed between the second (Day 30) and third (Day 31) days of acquisition, compared to what was observed at the start of training on Day 29. Importantly, also as observed in the plotted data, VHL-R and SH-R controls did not show differences between each other, even though only the VHL-R group received a VHL i.c.v. injection during acquisition, specifically after training on the third day of acquisition (Day 31). In contrast to our findings with the VHL and SH running versus control groups, C75 blocked the running-induced benefits in spatial learning seen in the VHL and SH groups (**Fig. 4** **,C**). There were no differences in escape latencies between C75-R and C75-S mice, although both groups did display significantly reduced latencies as the number training trials increased, indicating that animals in both groups learned during the task in spite of the absence of the cognitive enhancement effect of running (Two-Way RM ANOVA: Running Factor - F_(1, 255)_ = 3.227, *P*>0.05; Trials Factor -F_(15,255)_ = 3.794, ***P<0.0001; Interaction - F_(15, 255)_ = 1.569, *P*>0.05; Subject Matching -F_(17,255)_ = 3.3897, ***P<0.0001). Post-testing analysis did not detect any significant differences between the differences groups during any time throughout acquisition and LTM testing. Interestingly, inspection of the plotted data in **Fig. 4** **,C** suggests that, while C75-R mice displayed improvements in escape latency during the second (Day 30) and third (Day 31) day of acquisition, these subjects were unable to transfer the learned information into LTM, which was tested on Day 33. The results show that the five C75 i.c.v. injections given throughout the 30 Day running period of running, followed by an additional C75 i.c.v. injection after training of Day 3 of Barnes maze acquisition, block the running-associated improvements in learning and LTM. Overall, these findings support the notion that blocking FASN activity, which is potentially enhanced through the demonstrated exercise-induced increase in *fasn* gene expression (**Fig. 2** **,A**), impairs the cognitive enhancing effects of exercise on learning on memory. Moreover, these results also suggest that FASN might be an important player necessary for the cognitive enhancing effects of voluntary exercise.

### C75 and Searching Strategies

**Figure 5:**
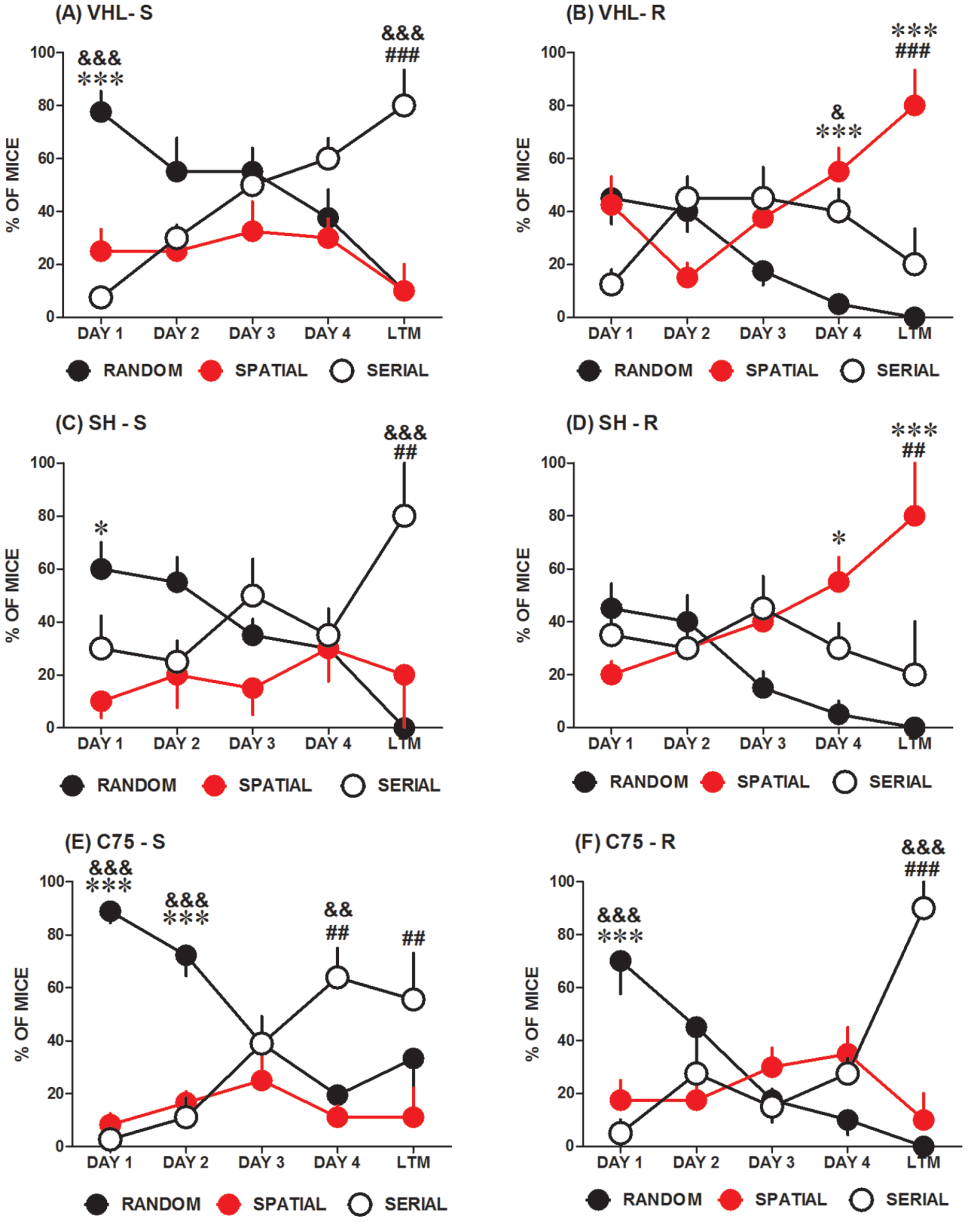
FASN inhibition in the forebrain affects escape latency and spatial discrimination in the Barnes maze. Graphed data depicting results obtained from acquisition training and LTM tests in the Barnes maze were given to Sedentary (punctuate line) and Running (solid line) mice injected with VHL (**A**), SH (**B**) or C75 (**C**). Each session during acquisition and LTM testing consisted of 4 training trials for which the latency in min to find the escape hole in the Barnes maze. Specific statistically significant differences between groups identified by Bonferroni post- testing specific are depicted (*P<0.05, **P<0.01, ***P<0.001). Results are presented as means ± SEM.

We next carried out specific analyses of the behavioral data in order to determine if animals in the different groups displayed differences in the usage of non-spatial (random), serial (thigmotaxic), or spatial searching strategies throughout acquisition and/or LTM testing. Results indicate that indeed animals in the various groups did display important differences with respect to the strategy used to search the maze during acquisition and LTM testing. Additionally, mice displayed a tendency to switch from random to either spatial or serial navigation strategies during acquisition. The VHL-S group (**Fig. 5** **,A**), showed a significant difference between the percent of mice using the different strategies on different days during acquisition and LTM testing (Two-Way RM ANOVA: Strategy - F_(2,108)_ = 6.886, **P<0.005; Training Days - F_(4,108)_ = 0.8978, *P*>0.05; Interaction - F_(8,108)_ = 8.251 ***P<0.0001). Bonferroni post-testing indicated that on Day 1 of acquisition training (day 29 in the study; see **Fig. 4**) there was a higher percentage of VHL-S mice searching randomly in the maze, compared to low percentages searching either serially (***P<0.001) or spatially (***P<0.001). However, as acquisition proceeded toward LTM testing, the serial strategy became preferred compared to the random (***P<0.001) and spatial strategies (***P<0.001).

For the VHL-R group (**Fig. 5** **,B**), the percentages of mice using the different strategies also changed throughout acquisition and LTM (Two-Way RM ANOVA: Strategy - F_(2,108)_ = 5.454, *P<0.05; Training Days - F_(4,108)_ = 0.000, *P*>0.05; Interaction - F_(8,108)_ = 9.197, ***P<0.0001). Bonferroni post-testing showed no significant differences in search strategy usage initially during acquisition. Importantly, the percentage of VHL-R mice searching spatially increased throughout acquisition, leading to significantly lower percentages of VHL-R mice searching randomly on Day 4 of acquisition (day 32 of the study; see **Fig. 4**) and during LTM testing (***P<0.001, respectively), the latter being also significantly lower in animals searching serially in the maze (***P<0.001). Interestingly, the usage of the serial strategy by VHL-R mice displayed a negative U shape curve, rather than the positive slope seen with the VHL-S group. In fact, the percent of VHL-R mice searching serially was significantly higher on Day 4 of acquisition than those searching randomly (*P<0.05). Such results suggest that the serial strategy was only of use to a small percentage of the VHL-R mice during the mid to final phases of acquisition occurring between Days 2 to 4 of training.

Similar to mice in the VHL-S group, SH-S mice (**Fig. 5** **,C**) displayed changes in the use of the different searching strategies throughout training and LTM testing (Two-Way RM ANOVA: Strategy - F_(2,48)_ = 4.990, *P<0.05; Training Days - F_(4,48)_ = 0.01187, *P*>0.05, Interaction - F_(8,48)_ = 3.964 **P<0.001). Bonferroni post-testing identified significantly more SH-S mice using the random strategy initially during acquisition, compared to those searching spatially (*P<0.05), which were similar in number to those SH-S mice searching serially (*P*>0.05). Interestingly, similar to VHL-S mice, serial navigation also became preferred for the SH-S mice compared to random (***P<0.001) and spatial searching (**P<0.01) during the LTM test, illustrating the switch in navigation modalities observed in all groups.

The SH-R group behaved similar to the VHL-R group (**Fig. 5** **,D**) with respect to navigational differences throughout acquisition and LTM testing (Two-Way RM ANOVA: Strategy - F_(2,48)_ = 6.662, *P<0.05; Training Days - F_(4,48)_ = 0.5079, *P*>0.05; Interaction - F_(8,48)_ = 3.975, **P<0.005). Initially, SH-R mice did not show significant differences with respect to the three searching strategies. However, most mice searched spatially on Day 4 (day 32 of the study; see **Fig. 4**), while a low percentage of mice searched either serially (*P<0.05) or randomly (*P*>0.05) at the same stage of acquisition, according to Bonferroni post-testing. During LTM testing (**Fig. 5** **,D**), most SH-R mice displayed spatial target zone navigation, compared to those searching serially (***P<0.001) or randomly (**P<0.01).

Results with the VHL-S vs. VHL-R and SH-S vs. SH-R are consonant with previous studies showing that running improves spatial learning and memory [12], [15]. In the case of the C75-S group (**Fig. 5** **,E**), on the other hand, mice displayed similar acquisition strategies as the VHL-S and SH-S cohorts (Two-Way RM ANOVA: Strategy - F_(2,96)_ = 10.93, ***P<0.0001; Training Days - F_(4,96)_ = 0.03719, *P*>0.05; Interaction - F_(8,96)_ = 9.615, ***P<0.0001). Bonferroni post-testing showed that more C75-S mice searched the maze randomly, compared to the number of animals using serial (***P<0.001) or spatial (***P<0.001) strategies on Days 1 and 2 (corresponding to days 29 and 30 of the study, respectively). However, as acquisition proceeded, more C75-S mice began to search serially, instead of randomly or spatially in the Barnes maze (**P<0.01, each comparison). C75-S mice still displayed the navigational divergence between serial and random strategies during LTM testing (**P<0.01). Importantly, no potential drug-related side effect affecting behavior could be attributed to C75, since C75-S mice behaved similarly in the maze as VHL-S and SH-S mice (**Fig. 5** **,A,C,E**).

Remarkably, and in agreement with our findings related to escape latencies (**Fig. 4**), the C75-R group yielded results that, while different to those seen in VHL-R and SH-R groups, were reminiscent to those observed in VHL-S and SH-S control groups (**Fig. 5A,B,C,D,F**). As opposed to the other two running groups and like all the Sedentary groups, C75-R mice (**Fig. 5** **,F**) began acquisition with a high preference for the random strategy and eventually displayed a high preference for the serial strategy upon LTM testing (Two-Way RM ANOVA: Strategy - F_(2,108)_ = 1.628, *P*>0.05; Training Days - F_(4,108)_ = 1.210, *P*>0.05; Interaction - F_(8,108)_ = 14.76, ***P<0.0001). According to post-testing analysis and similar to the mentioned behavior of Sedentary controls (**Fig. 5A,C**), most C75-R mice (**Fig. 5** **,F**) chose random navigation rather than either serial (***P<0.001) or spatial (***P<0.001) searching on Day 1 (day 29 of the study, **Fig. 1** **, Experiment 2**). Importantly however, in contrast to the results obtained in both VHL-R and SH-R groups (**Fig. 5** **,B,D,** respectively), C75-R mice did not display a learning-related increase in the use of the spatial strategy accompanied by a reduction in random navigation throughout acquisition training. Instead, as was the case for all the Sedentary groups (**Fig. 4A,C**), most C75-R mice (**Fig. 5** **,F**) preferred to search serially, rather than randomly or spatially, during LTM testing (***P<0.001). Taken together, our results recapitulate the beneficial effects of running on spatial learning and memory, and demonstrate that such cognitive enhancing effects of voluntary exercise are abolished by inhibition of FASN activity in the forebrain using i.c.v. C75.

### C75 and Spatial Discrimination LTM

**Figure 6:**
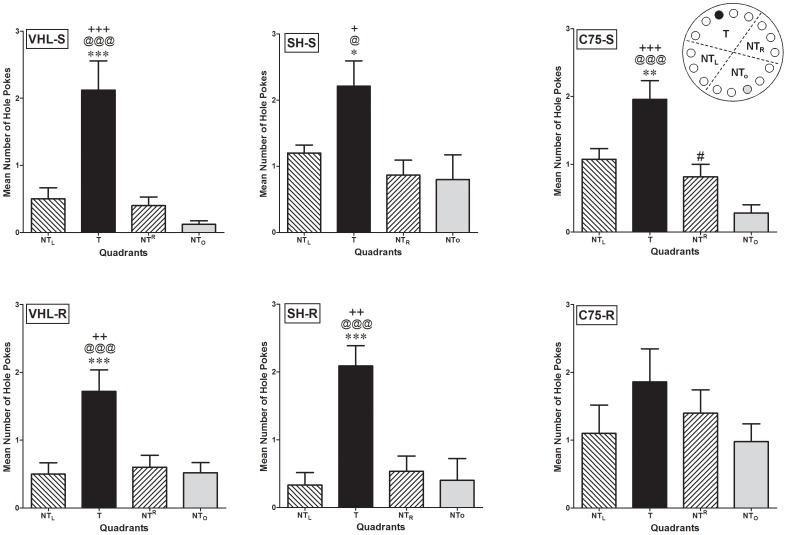
C75 affects LTM of spatial discrimination in the Barnes maze. Bar graphs depicting the preference of mice to poke in holes within the T, NT-L, NT-R, and NT-O quadrants of the Barnes maze. Data for each group were analyzed by One-Way ANOVA coupled to multiple post-testing analyses. Statistically significant differences between groups were observed according to the number of pokes each animal executed to find the target hole identified significant differences. Data are expressed as the mean number of hole pokes ± SEM. VHL-S, VHL-R, SH-S, SH-R, and C75-S, but not C75-R, displayed significant spatial discrimination between the T vs. NT-L (***P<0.001), NT-R (^+*++*^ *P*<0.01), and NT-O (@@@P<0.001) during the LTM test.

As seen in **Fig. 6** **,** we also analyzed the mean number of hole pokes in the four zones of the Barnes maze during our LTM test. The four zones were: i) the target zone (T) including the escape hole; ii) the non-target zone to the left of the T zone (NT-L), iii) the non-target zone to the right of the T zone (NT-R), and iv) the non-target zone opposite to the T zone (NT-O). In the case of the VHL-S (**Top Left**), SH-S (**Top Center**) and C75-S (**Top Right**) groups, One-Way ANOVA identified significant differences in the mean number of hole pokes to NT_L_, T, NT_R_, and NT_O_ zones (F_(3,39)_ = 13.81, ***P<0.0001; F_(3,36)_ = 13.56, ***P<0.0001, and F_(3,19)_ = 4.788, *P<0.0001 respectively). According to post-testing analysis, VHL-S, SH-S, and C75-S mice displayed a marked preference to visit the T zone compared to the other areas of the Barnes maze (VHL-S: NT-L vs. T, ***P<0.001; NT-R vs. T,^***^P<0.001; NT-O vs. T, ^***^P<0.001; SH-S: NT-L vs. T, *P<0.05; NT-R vs. T, ^*^P<0.05; NT-O vs. T, ^*^P<0.05; and C75-S: NT-L vs. T, ***P<0.001; NT-R vs. T, ^***^P<0.001; NT-O vs. T, ^**^P<0.001). These findings indicate that C75-S mice, reared in sedentary conditions, displayed no general impairment or toxicity in response to the treatment, compared to the other sedentary controls. One-Way ANOVA also identified significant differences in the mean number of hole pokes to NT_L_, T, NT_R_, or NT_O_ zones in VHL-R (**Bottom Left**) and SH-R (**Bottom Center**) mice (F_(3,39)_ = 7.67, ***P = 0.0004 and F_(3,19)_ = 10.12, ***P = 0.0006 respectively), with specific significant discrimination for the T over the NT-L (**P<0.001), NT-R (***P<0.001), and NT-O (***P<0.001) zones in both groups. In contrast to what was observed in the C75-S group and to the results obtained with VHL-R and SH-R mice, no significant zone discrimination was identified for the C75-R (**Bottom Right**) mice (F_(3,39)_ = 1.025, *P*>0.05). Since the LTM-impairing effects of C75 were only observed in the running and not the sedentary mice, these results substantiate the findings and conclusions obtained with the escape latencies and the analysis of the searching strategies (**Figs. 4** **,** **5**), supporting the notion that C75 treatment blocked the improvement in spatial learning and memory induced by running. Moreover, as mentioned above, since we showed that our running protocol induced FASN expression and the accumulation of its biosynthetic products PA and SA in a hippocampal-specific fashion, we propose that the effects observed for C75 are the result of blocking the running-induced function of FASN possibly ensuing in a lack of accumulation of the enzyme’s biosynthetic products.

### Chronic C75 Impairs Running-induced Proliferation in the SGZ of the DG

**Figure 7:**
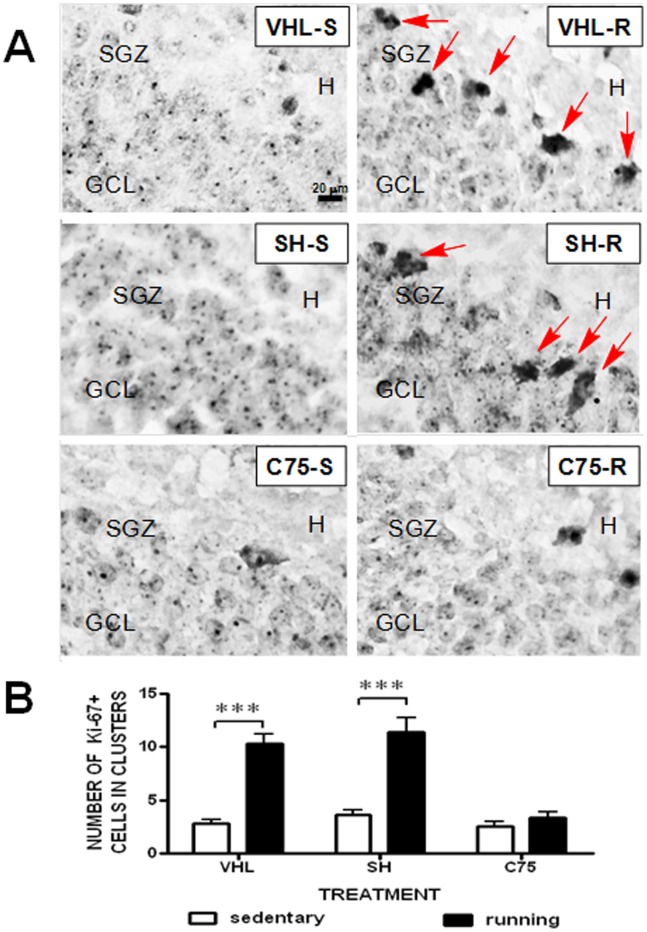
Immunohistochemistry for Ki-67+ in the SGZ of control versus treated mice. Injected sedentary (VHL-S, C75-S) and running (VHL-R, C75-R) groups (n = 6 per group), as well as both sham controls (SH-S and SH-R; n = 5 per group), were sacrificed at the end of the four weeks of the sedentary or running period of our experimental design and their brains were used for brain Ki-67+ immunohistochemistry. (**A**) Representative photomicrographs show characteristic Ki-67+ clusters of round to oval-shaped cells (arrows) in the SGZ of the DG were observed to be distributed randomly in both blades of the DG. GCL, granule cell layer; SGZ, subgranular zone; H, hilus. The scale bars represent 20 µm. (**B**) Comparison between the Sedentary and Running groups with different treatments: C75, VHL, and Sham. Bonferroni post-testing ***P<0.001 revealed statistically significant differences among the groups, indicating that running increased Ki-67+ labeling, an effect that was blocked by C75 treatment. Error bars indicate means ± SEM.

Since running has been shown to induce hippocampal neurogenesis [6], [12], [15], [17], [48], [49], [50], which in turn has been strongly implicated in learning and memory processes, and because of the known role of FASN in cellular proliferation, we hypothesized that blocking FASN function with C75 during our running protocol would also block hippocampal neurogenesis. We specifically asked if FASN inhibition interferes with cell proliferation by measuring Ki-67 immunohistochemistry in the SGZ of the DG of running mice. Assessment of Ki-67 was used instead of BrdU since it is an effective mitotic marker and has most of the benefits of BrdU as shown by Kee et al., (2002), [51]. In addition, multiple injections of BrdU may produce toxic effects to the animals and this would have added extra confounding side effects to our study. Animals in both VHL-R and SH-R groups, displayed Ki-67+ cells as scattered cluster formations, known to represent enhancement in cellular proliferation [52], within the SGZ both in the supra- and infrapyramidal blades of the rostral DG (**Fig. 7** **,A,B**). We analyzed the data by comparing the number of clusters of Ki-67+ cells in the DG SGZ region in the brains of mice from each group. Two-Way RM ANOVA analysis demonstrated that running increased the number of Ki-67+ cell clusters in the DG SGZ region in all groups expect the C75 treated animals (Treatment Factor: F_(2,14)_ = 31.45, ***P<0.0001; Running Factor: F_(1,14)_ = 57.35, ***P<0.0001; Interaction: F_(2,14)_ = 9.197, **P<0.002). According to Bonferroni post-testing, a higher number of Ki-67+ cell clusters were found in VHL-R and SH-R mice compared to their Sedentary controls (***P<0.0001, each comparison), whereas no specific differences in the number of Ki-67+ cell clusters were observed between C75 treated sedentary or running mice. In fact, the number of Ki-67+ cell clusters in the C75-R group was similar to that of all sedentary control groups (**Fig. 7**). These results suggest that blocking FASN function with C75, blocks the running-induced stimulation of hippocampal neurogenesis in 20 week-old mice.

## Discussion

Lipid metabolism is important for normal brain function and is relevant to neuropsychiatric and neurological disorders [20], [21]. Here we asked if voluntary exercise, which is known to enhance cognitive processes [6], [7], [11], [12], [13], [14], [15], [16], [17], [53], stimulates brain metabolism of bioactive lipids, including saturated and unsaturated FA, and whether biosynthetic machineries play an important role in cognition. Voluntary exercise is beneficial for brain plasticity, stimulates neurogenesis, as well as neuroadaptive, and neuroprotective responses [6], [7], [11], [12], [13], [14], [15], [16], [17], [53]. However, the metabolic pathways potentiated by physical activity in the brain that may be associated to these processes are poorly understood. In a distinct set of studies (Santos-Soto et al., unpublished observations), we reported that running induces a FA profile in the brains of 8 week–old young adult male mice, different than the one reported here for the 20 week-old male mice. Specifically, young adult mice responded to exercise by increasing cortical of docosahexaenoic acid (DHA, omega 3-C22∶6) and arachidonic acid (AA, omega 6-C20∶4), while decreasing PA levels in the same region. Unlike, the studies presented here, no changes were observed in the hippocampus with respect to FA profiling. These differences observed between running-induced FA profiling in young adult versus 20 week-old mice are probably reflective of age-dependent differences in molecular and biochemical plasticity ruling the response of the brain to exercise.

Our findings revealed that voluntary running results in upregulation of *fasn* mRNA in the mouse hippocampus, yet not in cortex or cerebellum (**Fig. 2A**). Voluntary running also caused hippocampal-specific accumulation of two known FASN biosynthetic products, PA and SA (**Fig. 2** **,B;** **Fig. 3**). The FASN pathway utilizes acetyl CoA and malonyl CoA to elongate FA by two carbons to initially produce PA in the cytosol [23], [54]. It is known that some amounts of SA may sometimes be formed through the action of FASN from PA [23], [55], while in most cases SA biosynthesis occurs via a different mechanism, that is, the elongation by FA elongases, ELOVL1 or ELOVL6 [23], [56]. Both ELOV-1/6 carry out the same reaction as FASN and are also regulated transcriptionally [23], [38]. FASN is expressed ubiquitously throughout the brain [57], ELOVL1 is mainly expressed in the corpus callosum, and ELOVL6 has relatively low brain expression, compared to the liver [56]. However, these FA biosynthetic enzymes differ in that FASN operates in the cytoplasm, while ELOV-1/6 function in the mitochondria and endoplasmic reticulum utilizing an enoyl-CoA reductase plus acetyl CoA and fatty acyl CoA as substrates [23], [38]. In order to determine if our findings with FASN were specific, or whether they could also apply to ELOV-1/6, we also examined the expression of ELOV-1/6 in our experimental paradigm. Our findings show that, opposite to FASN, ELOV-1/6 mRNA expression was not significantly affected by running in any of the regions tested (**Fig. 2** **,C,D**). Since no changes were observed in *elovl-1/6* mRNA levels in all studied brain regions, we conclude that FASN, and not ELOVL-1/6, is possibly responsible for the hippocampal-specific increases in both PA and SA induced by running, the latter through *de-novo* chain elongation from PA (see **Figs. 2** **,A, B, &** **Fig. 3**).

### Roles of PA and SA as Potential Bioactive Lipids in Learning and Memory

Disrupting PA and SA biosynthesis via inhibition of FASN function (see below), may alter intracrine or paracrine mechanisms potentially relevant for hippocampal synaptic plasticity, learning and memory. As the most common saturated FA, being the predominant *de novo* product of FASN, PA is known for its role in axon myelination, axonal transport, and vesicular packaging and release [58], each being essential neurochemical mechanisms involved in basal neuronal function that are also required for neurotransmission, plasticity, learning, and memory. PA is a common substrate for posttranslational modification of proteins via palmitoylation [59], which modulates protein flexibility within cell membranes and synapses resulting in enhanced synaptic communication and cell signaling [60], [61], [62]. In particular, PA modulates the trafficking of N-methyl-D-aspartate (NMDA) receptor subunit, an effect that might be important for NMDA-receptor-dependent synaptic plasticity [63]. Palmitoylation plays a key role in synapse stabilization by aiding the trafficking of postsynaptic density protein 95 (PSD95) and glutamate receptors, including the α-amino-3-hydroxy-5-methyl-4-isoxazolepropionic acid receptor (AMPA-R), specifically in the trans-membrane domain of the C terminus of its subunits, and the NMDA receptor, specifically on its NR2 subunits [59], [63]. In addition, palmitoylation of PSD95 is increased by brain derived neurotrophic factor (BDNF) stimulation through the PI3 kinase-Akt pathway downstream of BDNF/TrkB signaling, which triggers synaptic delivery of palmytoilated PSD95 via vesicular transport [64]. It is recognized, that increased expression of BDNF and other growth factors by voluntary running plays a critical role in neuronal survival and differentiation [5]. Thus, the hippocampal upregulation of FASN expression, observed by us here, and that of BDNF, reported by others [5] as a result of exercise, could be mechanistically associated to a FASN-driven machinery for enhanced palmitoylation, which in concert with BDNF may promote synaptic plasticity and facilitate learning and memory.

With respect to SA, this saturated FA has been shown to be neuroprotective [19] and to act as an endogenous ligand of PPARs, which are ligand-dependent transcription factors regulating lipid homeostasis [65]. Interestingly, PPAR agonists have been shown in clinical trials to improve memory and cognition in Alzheimer’s disease. Specifically, the PPARγ agonist, rosiglitazone, improves cognition and protects from neuropathology associated to Alzheimer’s disease in transgenic mouse models and in humans [66].

The direct impact of PA or SA on hippocampal memory function has not been completely studied, except via indirect experiments involving high-fat diet [67]. In one study, infusions of PA into the third ventricle were of no effect with respect to memory in the T-maze foot-shock avoidance test in mice [68]. Such lack of effects on cognition might be due to the dilution of PA within the cerebral ventricles. Based on the findings obtained here, the study of the role of PA or SA on learning and memory should require local infusions within discrete brain regions, rather than ventricle microinjections in order to avoid confounding factors, such as a dilution throughout the cerebrospinal fluid as well as the forebrain parenchyma.

On the other hand, PA and SA can also be converted into the endogenous endocannabinoid-like molecules, N-acyl-ethanolamines: N-palmitoylethanolamide (PEA) and N-stearoylethanonamide (SEA), respectively, via biochemical reactions with ethanolamide intracellularly [69], [70]. Both PEA and SEA are degraded by intracellular serine FA amide hydrolase (FAAH, [71], [72]). Interestingly, FAAH inhibition enhances learning and memory [69], perhaps by leading to increased PEA and SEA levels. In addition, PEA has a protective effect on hippocampal DG granule cells by acting as a ligand to PPARα, a member of a family of ligand-dependent transcription factors regulating lipid homeostasis that has also been associated to learning and memory [65].

### Forebrain Inhibition of FASN Impairs Spatial Learning and Memory

Our findings obtained with respect to exercise, that is, FASN hippocampal upregulation with concomitant hippocampal FA accumulation in the present studies, lead us to further examine the role of FASN in voluntary running associated processes: specifically cognitive enhancement. We tested whether FASN function is required for hippocampal-dependent memory, a processes potentiated by running [12], [15], by inhibiting forebrain activity of FASN using i.c.v. injections of C75, a specific irreversible antagonist of the enzyme. C75 inactivates the beta-ketoacyl synthase, enoyl reductase and thioesterase partial activities of FASN [41]. Central administration of C75 causes profound dose-dependent anorexia and weight loss in mice via alteration of neuronal energy balance by elevation of hypothalamic neuronal ATP levels, which eventually leads to a decrease in hypothalamic neuropeptide Y levels [43].

Assessment of spatial learning and memory revealed improved escape latencies in VHL-R vs. VHL-S mice and in SH-R vs. SH-S mice confirming the beneficial effects of voluntary running on hippocampal-dependent learning and memory [5], [12], [15]. Importantly, C75-S mice behaved similarly in the Barnes maze as VHL-S and SH-S animals, a finding that supports the notion that the drug had no toxic effects that in itself impaired learning the task. Remarkably, however, C75-R mice showed significantly delayed escape latencies in the Barnes maze, suggesting that inactivating FASN considerably impaired learning and memory (**Fig. 4**). Furthermore, the fact that the VHL and SH, but not C75, running groups displayed improved spatial learning performance and discrimination memory, compared to their respective Sedentary controls **(** **Figs. 4** **,** **6** **)**, strongly suggests that C75 blocked the cognitive enhancing effects of exercise.

The data from the distinct searching strategies used by animals during Barnes maze acquisition and consolidation also supported the findings discussed so far (**Fig. 5**). When comparing the use of search strategies during the LTM tests, the data suggest that i) running increases the use of spatial navigation, while simultaneously reducing the use of both random and serial search strategies in the Barnes maze; and that ii) such running-induced beneficial effect on spatial learning is blocked by C75 treatment, as evidenced by the observation of mostly serial navigation by the C75-R group, a non-spatial searching strategy predominant in all Sedentary controls. This finding is relevant *in lieu* of the fact that serial, or thigmotaxic, memories can be retained for at least 24 h, but are not consolidated [73] into more enduring memories. In summary, C75 impaired spatial searching during the LTM test causing treated animals to resort to the non-spatial serial searching strategy, which does not result in long-lasting memory [66], as a compensatory mechanism for escaping from the maze. Interestingly, different neural substrates such as those in the striatum [74], are known to be important for serial searching strategies. Finally, the results from the spatial discrimination test indicate that VHL-S, VHL-R, SH-S, SH-R, and C75-S, but not C75-R, displayed significant spatial discrimination favoring navigation within the T quadrant of the Barnes maze during the LTM test. The fact that C75-S, and not C75-R, mice developed appropriate spatial discrimination is consistent with our findings showing that the C75-S group did express learning and memory on the Barnes maze with respect to the observed mean escape latencies (**Fig. 4**). Moreover, that C75-S mice were capable of learning and forming LTM, unlike the C75-R group, suggests that voluntary running induces a different mechanism for learning and memory than the one utilized under sedentary conditions. Specifically, we postulate that running induces a hippocampal-specific FASN-dependent pathway that results in cognitive enhancement. Importantly, since running is associated with hippocampal neurogenesis, such FASN-dependent pathway may be specifically associated to stimulation of processes related to cellular proliferation in the hippocampal formation. The fact that Sedentary mice treated with C75 were capable of learning and of forming LTM, while Running animals could not, suggests that exercise activates the proposed hippocampal-specific FASN-dependent pathway, which conceivably functions in substitution of cellular and molecular pathways acting under sedentary conditions.

### Blocking Forebrain FASN Function With C75 Impairs Running-Induced Cellular Proliferation in the Hippocampus

Exercise is known to increase neurogenesis in the hippocampal formation [6], [12], [15], [48], [49], [50]. Such neurogenesis is initiated by the proliferation of progenitor type-2a/b and type-3 cells that cluster symmetrically within the SGZ of the supra- and infrapyramidal blades of the rostral DG [48], [75]. Importantly, running also profoundly induces differentiation of progenitors into neurons, rather than into glia [12].

Consistent with previous reports, we found more proliferative Ki-67+ cells clustered at the border of the granular cell layer and the hilus within the SGZ of VHL-R and SH-R groups compared to sedentary VHL-S, SH-S, and C75-S controls (**Fig. 7**). On the one hand, since the DG region of the hippocampus of Running mice has more new neurons than that of Sedentary mice [6], [12], [15], [48], [49], [50], the detected over expression of FASN in our studies may be in part relevant to the elevation of a population of neurons that express FASN more abundantly [76]. Hence, regional dissection of the hippocampus and the DG into subfields in order to clarify the subregional distribution of FASN induction will be addressed in future studies. On the other hand, since FASN is essential for cellular proliferation [22], [26], [57], the observed enhanced cellular proliferation that occurred in tight clusters in the DG [52] may be a result of the running-induced enhanced expression and activity of FASN. This latter assumption is supported by the results presented in **Fig. 7** depicting evidence that C75-R groups displayed reduced cell proliferation, compared to VHL-R/SH-R controls, indicating that such treatment also impaired running-induced hippocampal neurogenesis.

In our studies, we examined Ki-67 immunoreactivity in animals that had undergone running for 33 days, the last five of which they were also learning in the spatial Barnes maze. Thus, it could be argued that the observed changes in DG cell proliferation could be due, perhaps in part, to spatial learning. It has been found that hippocampal-dependent learning can enhance proliferation of hippocampal cells [77]. However, while several studies have suggested that blocking hippocampal neurogenesis impairs learning and memory, the notion of whether learning activity induces dentate gyrus neurogenesis is still controversial [78]. van Praag et al. (1999) [6] reported that when subjecting mice to spatial training in the Morris water maze for 30 days, no increases in DG neurogenesis were observed. Similar findings were obtained by Van der Borght et al. [17]. Other studies have actually reported a decrease in DG cell proliferation as a result of hippocampal dependent learning [79]. The differential effects of learning on DG cell proliferation versus survival of newborn neurons has been suggested in studies obtaining contradicting effects in terms of enhancement or decrease of new neurons in response to hippocampal-dependent learning, in some cases observing a diverting effect dependent on the stage of acquisition [80], [81]. Taken together, these studies lead to the conclusion that it would be too preliminary to attempt to suggest that the enhancement in DG proliferation reported by us here is associated to learning, although running-induced neurogenesis itself may have been responsible for improved learning and memory in running animals.

The fact that running induces neurogenesis is very well established in the literature [6], [12], [15], [48], [49], [50]. As mentioned earlier, FASN is essential for cellular proliferation [22], [26], [57]. Thus, our studies in **Experiment 2 (** **Fig. 1** **)** were designed to determine whether blocking FASN activity with C75 blocked DG proliferation induced by running. Actually, it is important to mention that during the 28-day running-only period in **Experiment 2**, mice received chronic injections of C75, while receiving only a single injection of C75 during Barnes maze training at the end of day 3 of acquisition. Since training and LTM testing only lasted for five days, it is more likely that the observed inhibitory effects of C75 on DG cell proliferation were related to its administration during the running period and not to its single dosing during the acquisition of the Barnes maze.

Overall, our findings support a role of FASN in the biochemical mechanisms subserving the raise of mitosis rates in the DG in response to voluntary running, which is in agreement with previous reports observing high FASN activity in adult proliferative cells [22], [82]. Finally, the fact that C75 blocked the benefits of running on cognitive performance suggests that the inhibitory effects of C75 on the proliferation of progenitors cells of the hippocampal DG in running mice may be at least partly associated to the lack of hippocampal-dependent cognitive gain observed in the exercised mice also treated with this FASN inhibitor (**Fig. 4** **,** **5** **,** **6**).

## Conclusion

Overall, our study suggests that running enhances the expression of FASN and the accumulation of specific saturated FA species, PA and SA, in the hippocampus, but not the cerebellum or cortex of 20 week-old male mice. The findings presented here also suggest that such biochemical changes are associated to the enhancement of hippocampal-dependent spatial learning and memory, as well as the stimulation of cellular proliferation in the DG, in response to physical exercise. Inhibition of FASN activity and function, as we did here using C75, may alter intracrine or paracrine mechanisms potentially relevant for the processes stimulated by running such as neurogenesis, hippocampal synaptic plasticity, and learning and memory. Overall, we conclude that FASN is an essential factor mediating the cognitive enhancement and hippocampal neurogenesis induced by exercise, potentially via the specific action of selected FA such as PA and SA.
